# Incidence of *Pneumocystis jirovecii* pneumonia utilizing a polymerase chain reaction‐based diagnosis in patients receiving bendamustine

**DOI:** 10.1002/cam4.4067

**Published:** 2021-06-22

**Authors:** Mikhaila L. Rice, Jason N. Barreto, Carrie A. Thompson, Kristin C. Mara, Pritish K. Tosh, Andrew H. Limper

**Affiliations:** ^1^ Department of Pharmacy Mayo Clinic Rochester MN USA; ^2^ Division of Hematology Department of Internal Medicine Mayo Clinic Rochester MN USA; ^3^ Division of Biomedical Statistics and Informatics Department of Health Sciences Research Mayo Clinic Rochester MN USA; ^4^ Division of Infectious Diseases Department of Internal Medicine Mayo Clinic Rochester MN USA; ^5^ Division of Pulmonary and Critical Care Medicine Department of Internal Medicine Mayo Clinic Rochester MN USA

**Keywords:** chemotherapy, clinical management, hematological cancer, lymphoma

## Abstract

**Background:**

*Pneumocystis jirovecii* pneumonia (PJP) is a life‐threatening infection occurring in patients receiving bendamustine. The poorly defined incidence, particularly when utilizing polymerase chain reaction (PCR)‐based diagnostic techniques, precipitates unclear prophylaxis recommendations. Our objective was to determine the cumulative incidence of PJP diagnosed by single copy target, non‐nested PCR in patients receiving bendamustine.

**Methods:**

Patients were evaluated for PJP from initiation of bendamustine through 9 months after the last administration. The cumulative incidence of PJP was estimated using the Aalen–Johansen method. Cox proportional hazard models were used to demonstrate the strength of association between the independent variables and PJP risk.

**Results:**

This single‐center, retrospective cohort included 486 adult patients receiving bendamustine from 1 January 2006 through 1 August 2019. Most patients received bendamustine‐based combination therapy (n = 461, 94.9%), and 225 (46.3%) patients completed six cycles. Rituximab was the most common concurrent agent (n = 431, 88.7%). The cumulative incidence of PJP was 1.7% (95% CI 0.8%–3.3%, at maximum follow‐up of 2.5 years), after the start of bendamustine (n = 8 PJP events overall). Prior stem cell transplant, prior chemotherapy within 1 year of bendamustine, and lack of concurrent chemotherapy were associated with the development of PJP in univariate analyses. Anti‐*Pneumocystis* prophylaxis was not significantly associated with a reduction in PJP compared to no prophylaxis (HR 0.37, 95% CI (0.05, 3.04), *p* = 0.36).

**Conclusions:**

Our incidence of PJP below 3.5%, the conventional threshold for prophylaxis implementation, indicates routine anti‐*Pneumocystis* prophylaxis may not be necessary in this population. Factors indicating a high‐risk population for targeted prophylaxis require further investigation.

## Graphical abstract

In this retrospective cohort of patients receiving bendamustine‐based therapy, the cumulative incidence of *Pneumocystis jirovecii* pneumonia was 1.7%, which falls below the accepted 3.5% threshold for universal prophylaxis. Anti‐*Pneumocystis* prophylaxis was not significantly associated with a reduction in PJP compared to no prophylaxis; however, defining the highest risk population to potentially benefit from a targeted prophylaxis approach requires further investigation.

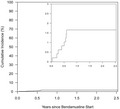

**LAY SUMMARY**

*Pneumocystis jirovecii* pneumonia (PJP) is a life‐threatening infection occurring in patients receiving bendamustine and the incidence, especially when attempting diagnosis with a new technology, remains unknown. Our retrospective study showed a 1.7% cumulative incidence of PJP in patients receiving bendamustine‐based therapy, which is below the accepted 3.5% threshold for universal prophylaxis. Prior stem cell transplant, prior chemotherapy within 1 year of bendamustine initiation, and lack of concurrent chemotherapy were associated with the development of PJP. Anti‐*Pneumocystis* prophylaxis was not significantly associated with a reduction in PJP compared to no prophylaxis.

## 1 INTRODUCTION

*Pneumocystis jirovecii* pneumonia (PJP) is a severe, opportunistic infection that has historically been found in patients who are infected with human immunodeficiency virus (HIV) or who are immunosuppressed with high doses of corticosteroids.^1^ PJP has been increasingly identified in patients with malignancies, especially hematological malignancies, who are prescribed immunosuppressive chemotherapy regimens.^1^, ^2^ HIV seronegative patients who develop PJP, especially with a cancer diagnosis, tend to have higher mortality (30%–60%) compared to those infected with HIV (10%–20%).^3^, ^4^, ^5^

Prophylaxis regimens for PJP are well established for HIV‐infected patients with T‐helper cell count (CD4+) less than 200 cells/mm^3^ and for patients taking high doses of corticosteroids; however, the indication for anti‐*Pneumocystis* prophylaxis is less clear in other immunosuppressed patient populations.^2^, ^6^ One meta‐analysis suggests initiating prophylaxis when risk for PJP is greater than 3.5%, a threshold which has been widely adapted for PJP research and included in antimicrobial prophylaxis guidelines.^7^, ^8^ Others have recommended routine anti‐*Pneumocystis* prophylaxis be provided when the incidence of PJP is 3–5% or higher.^9^

Bendamustine is a nitrogen mustard derivative with alkylating properties that was initially approved by the FDA in 2008 for treatment of chronic lymphocytic leukemia (CLL).^10^, ^11^ Efficacy demonstrated in the treatment of other hematologic malignancies has propagated use as frontline, second‐line, or salvage therapy.^12^ Reports describing PJP development in patients on bendamustine therapy appeared as early as 2003.^13^, ^14^, ^15^, ^16^, ^17^, ^18^, ^19^ Bendamustine has been shown to decrease CD4+ counts, with return to baseline levels taking as long as 7–9 months following the completion of treatment.^15^, ^16^, ^18^, ^20^ Despite these findings, there is no clear recommendation for anti‐*Pneumocystis* prophylaxis for patients receiving bendamustine.^8^, ^21^, ^22^ A recent Surveillance, Epidemiology, and End Results (SEER)–Medicare cohort study of over 1,200 patients with indolent Non‐Hodgkin lymphomas reported that those treated with bendamustine had a significantly increased risk of opportunistic infections, although PJP risk was only significantly increased when bendamustine was used as a third‐line therapy (HR 3.32, 95% CI 1.00‐11.11).^23^

Recommendations for anti‐*Pneumocystis* prophylaxis in patients on bendamustine therapy rely on an appropriately defined incidence using contemporary diagnostic techniques. PJP was historically diagnosed by respiratory sample analysis via Giemsa or Giemsa‐like rapid stains, Gomori methenamine silver stain, toluidine blue O stain, and fluorescein‐conjugated monoclonal antibody (direct fluorescent‐antibody [DFA] stain).^2^ Techniques used in the systematic review and meta‐analysis that helped define the 3.5% threshold for prophylaxis relied upon microbiological or histopathological evaluation of respiratory samples or lung biopsies with some included studies utilizing direct demonstration by monoclonal antibodies or silver stain.^7^ However, PCR‐based techniques for respiratory sample analysis have demonstrated improved sensitivity compared to other methods, and the use of positive *Pneumocystis* PCR in conjunction with clinical evaluation has become the more common method of diagnosis.^24^, ^25^, ^26^, ^27^ The purpose of this study is to determine the incidence of PJP diagnosed by single copy target, non‐nested PCR in patients receiving bendamustine. Results from this investigation will guide future decision‐making surrounding anti‐*Pneumocystis* prophylaxis in this patient population.

## 2 METHODS

This is a retrospective, observational cohort study of adults aged 18 years and older who received at least one dose of bendamustine between 1 January 2006 and 1 August 2019 at a large academic health center. Patients were excluded if they were HIV seropositive, refused research consent, or were considered part of a vulnerable population. The study was reviewed by the local Institutional Review Board (IRB) and determined to be exempt from the requirement for IRB approval (IRB #19‐007217, Approval Date: 20 September 2019). After inclusion, an electronic health record (EHR) review was performed in accordance with the ethical standards of the 1964 Declaration of Helsinki with adherence to all relevant regulations of the US Health Insurance Portability and Accountability Act.

Demographic data, laboratory values, cancer diagnosis, medication administrations, PJP diagnosis with supporting *Pneumocystis* PCR and radiographic imaging results, and patient outcomes were abstracted from the patient EHR utilizing a standardized data collection form and managed with REDCap (Research Electronic Data Capture).^28^ Cancer diagnosis with subtyping was according to World Health Organization (WHO) classification criteria and prior to systemic chemotherapy.^29^ Diagnosis of PJP was collected from initiation of bendamustine through 9 months after the last dose of bendamustine. A positive *Pneumocystis* PCR using our single copy gene‐targeted, non‐nested, real‐time PCR assay from a respiratory sample in conjunction with clinical signs and symptoms of pulmonary compromise on medical record review constituted a diagnosis of PJP. The detection threshold of this PCR diagnostic assay was set to detect active PJP, minimizing potential false positives caused by *Pneumocystis* colonization, and with overall sensitivity exceeding 96%.^24^, ^26^ The initial development and the quantitative PCR methods and conditions were described previously and set a quantitative threshold at 45 cycles.^24^, ^26^

Data pertaining to patient demographics and clinical characteristics were summarized with descriptive statistics. These included counts and percentages for categorical and ordinal variables and medians and interquartile ranges (IQR) for continuous variables. The cumulative incidence of PJP was estimated using the Aalen–Johansen method where death was a competing risk. Cox proportional hazards regression was used to demonstrate the strength of association between the independent variables and risk of PJP. These associations were summarized with hazard ratios (HR) and 95% confidence intervals (CI). All analyses were carried out using SAS (Version 9.4, SAS Institute Inc., Cary, NC). A *P* value less than 0.05 was considered statistically significant.

## 3 RESULTS

A total of 486 patients met inclusion criteria and were included in the analysis. The median patient age was 66 years (range 20–94) and 294 (60.5%) were male. Patients were mostly Caucasian (n = 457, 94.0%). The most common diagnosis was follicular lymphoma (n = 180, 37.0%) according to WHO classification criteria. Additional baseline demographic and laboratory information for the study population is summarized in Table 1.

**TABLE 1:** Baseline characteristics and laboratory values

Characteristic	Patients with PJP (n = 8)	Patients without PJP (n = 478)	Total (n = 486)
Age, years, median (range)	69 (36–76)	66 (20–94)	66 (20–94)
Male, No. (%)	4 (50.0)	290 (60.7)	294 (60.5)
Race, No. (%)			
Caucasian	8 (100.0)	449 (93.9)	457 (94.0)
Other	0 (0.0)	29 (6.1)	29 (6.0)
Body Surface Area, m^2^, median (IQR)	2.04 (1.72–2.23)	1.97 (1.79–2.15)	1.97 (1.79–2.15)
Body Mass Index, kg/m^2^, median (IQR)	27.0 (23.9–29.7)	27.2 (23.8–31.4)	27.2 (23.8–31.4)
Cancer Diagnosis, No. (%)			
Follicular lymphoma	3 (37.5)	177 (37.0)	180 (37.0)
Mantle cell lymphoma	1 (12.5)	77 (16.1)	78 (16.0)
Marginal zone lymphoma	0 (0.0)	55 (11.5)	55 (11.3)
Diffuse large B‐cell lymphoma	0 (0.0)	44 (9.2)	44 (9.1)
Waldenström macroglobulinemia	0 (0.0)	41 (8.6)	41 (8.4)
Chronic lymphocytic leukemia/small lymphocytic lymphoma	0 (0.0)	21 (4.4)	21 (4.3)
Other	4 (50.0)	63 (13.2)	67 (13.8)
Phase of Diagnosis ‐ Primary, No. (%)	8 (100.0)	440 (92.1)	448 (92.2)
Charlson Comorbidity Index, median (range)	5.5 (4–13)	5 (0–19)	5 (0–19)
COPD, No. (%)	0 (0.0)	40 (8.4)	40 (8.2)
CKD, No. (%)	1 (12.5)	74 (15.5)	75 (15.4)
ESLD, No. (%)	0 (0.0)	2 (0.4)	2 (0.4)
Solid Organ Transplant, No. (%)	0 (0.0)	3 (0.6)	3 (0.6)
Stem Cell Transplant, No. (%)	3 (37.5)	48 (10.0)	51 (10.5)
Autologous	2 (25.0)	43 (9.0)	45 (9.3)
Allogeneic	1 (12.5)	5 (1.0)	6 (1.2)
Autoimmune Disease, No. (%)	1 (12.5)	19 (4.0)	20 (4.1)
Rheumatoid Arthritis	1 (12.5)	9 (1.9)	10 (2.1)
Systemic lupus erythematosus	0 (0.0)	7 (1.5)	7 (1.4)
Inflammatory Bowel/Crohn’s Disease	0 (0.0)	4 (0.8)	4 (0.8)
WBC count, ×10^9^/L, median (IQR)	7.5 (4.7–9.7)	6.5 (4.9–9.6)	6.5 (4.8–9.6)
Hgb, g/dL, median (IQR)	10.2 (9.9–11.6)	12.0 (9.9–13.5)	12.0 (9.9–13.5)
ANC, ×10^9^/L, median (IQR)	4.81 (3.35–5.29)	3.82 (2.65–5.15)	3.82 (2.66–5.16)
ALC, ×10^9^/L, median (IQR)	1.15 (0.72–1.55)	1.17 (0.71–1.85)	1.17 (0.71–1.84)
Serum Creatinine, mg/dL, median (IQR)	1.1 (0.8–1.1)	1.0 (0.8–1.2)	1.0 (0.8–1.2)
Estimated Creatinine Clearance, No. (%)			
>60 mL/min	5 (62.5)	304 (65.2)	309 (65.2)
31–60 mL/min	3 (37.5)	138 (29.6)	141 (29.7)
<31 mL/min	0 (0.0)	24 (5.2)	24 (5.1)
BUN, mg/dL, median (IQR)	18 (18–19)	18 (13–26)	18 (13–25)
ALT, units/L, median (IQR)	21 (19–23)	21 (16–32)	21 (16–31)
AST, units/L, median (IQR)	25 (21–31)	25 (20–34)	25 (20–34)
ALP, units/L, median (IQR)	79 (60–105)	81.5 (66–104)	81.5 (66–104)
Total Bilirubin, mg/dL, median (IQR)	0.7 (0.4–0.9)	0.5 (0.3–0.7)	0.5 (0.3–0.7)
Direct Bilirubin, mg/dL, median (IQR)	0.3 (0.3–0.3)	0.2 (0.1–0.3)	0.2 (0.1–0.3)
Albumin, g/dL, median (IQR)	3.5 (3.5–3.6)	3.5 (3.1–3.9)	3.5 (3.1–3.8)
LDH, units/L, median (IQR)	233 (202–278)	206 (168–278)	206 (168–278)

Abbreviations: ALC, absolute lymphocyte count; ALP, alkaline phosphatase; ALT, alanine transaminase; ANC, absolute neutrophil count; AST, aspartate transaminase; BUN, blood urea nitrogen; CI, confidence interval; CKD, chronic kidney disease; COPD, chronic obstructive pulmonary disease; ESLD, end stage liver disease; IQR, interquartile range; LDH, lactate dehydrogenase; PJP, Pneumocystis jirovecii pneumonia; WBC, white blood cell.

Bendamustine was provided as first‐line therapy in 297 (61.1%) patients, second‐line in 68 (14.0%) patients, and salvage beyond second‐line in 121 (24.9%) patients. The majority of patients (n = 461, 94.9%) received bendamustine‐based combination therapy, and 25 (5.1%) patients received bendamustine monotherapy. Detailed information about the bendamustine‐based chemotherapy regimens prescribed is available in Table S1. The most common treatment administered in conjunction with bendamustine was rituximab (n = 431, 88.7%). A total of 225 (46.3%) patients completed six cycles of bendamustine with 215 (44.2%) patients receiving 12 doses of bendamustine. The median cumulative dose of bendamustine was 805 mg/m^2^ (IQR 437–1080). Steroids were prescribed to 377 (77.6%) patients during bendamustine therapy, and 13 (2.7%) patients received additional immunosuppressive medications including: sirolimus (n = 4), tacrolimus (n = 4), cyclosporine (n = 3), mycophenolate mofetil (n = 3), everolimus (n = 2), and azathioprine (n = 1). In the 9‐month post‐bendamustine observation, 175 (36.0%) patients received additional therapy for treatment of their malignant disease; 78 (44.6%) of these patients received rituximab maintenance. Additional detail regarding chemotherapy and immunotherapy use is provided in Table 2.

**TABLE 2:** Chemotherapy characteristics and medication use

Characteristics	Patients with PJP (n = 8)	Patients without PJP (n = 478)	Patients (n = 486)
Line of chemotherapy, No. (%)			
1	4 (50.0)	293 (61.3)	297 (61.1)
2	0 (0.0)	68 (14.2)	68 (14.0)
≥3	4 (50.0)	117 (24.5)	121 (24.9)
Prior chemotherapy within one year, No. (%)	4 (50.0)	118 (24.7)	122 (25.1)
Chemotherapy most immediately prior to bendamustine within 1 year, No. (%)			
R‐CHOP	0 (0.0)	14 (11.9)	14 (11.5)
Rituximab monotherapy	0 (0.0)	13 (11.0)	13 (10.7)
R‐CVP	0 (0.00)	5 (4.2)	5 (4.1)
Rituximab and methylprednisolone	0 (0.0)	5 (4.2)	5 (4.1)
Other	4 (100.0)	81 (68.6)	85 (69.7)
Cumulative bendamustine dose, mg/m^2^, median (IQR)	650 (450–1010)	810 (437–1080)	805 (437–1080)
Chemotherapy cycles, median (range)	4.5 (1–6)	5 (1–10)	5 (1–10)
Chemotherapy cycles, No. (%)			
1	2 (25.0)	64 (13.4)	66 (13.6)
2	0 (0.00)	46 (9.6)	46 (9.5)
3	2 (25.0)	47 (9.8)	49 (10.1)
4	0 (0.0)	68 (14.2)	68 (14.0)
5	0 (0.0)	26 (5.4)	26 (5.3)
≥6	4 (50.0)	227 (47.5)	231 (47.5)
Concurrent chemotherapy, No. (%)	4 (50.0)	457 (95.6)	461 (94.9)
Rituximab	3 (37.5)	428 (89.5)	431 (88.7)
Bortezomib	0 (0.0)	13 (2.7)	13 (2.7)
Brentuximab	0 (0.0)	10 (2.1)	10 (2.1)
Lenalidomide or thalidomide	0 (0.0)	10 (2.1)	10 (2.1)
Other	1 (12.5)	24 (5.0)	25 (5.1)
Steroids received, No. (%)	8 (100.0)	369 (77.2)	377 (77.6)
Receipt of additional chemotherapy within nine months after completion of bendamustine, No. (%)	4 (50.0)	171 (35.8)	175 (36.0)
Rituximab monotherapy	1 (25.0)	77 (45.0)	78 (44.6)
Other	3 (75.0)	94 (55.0)	97 (55.4)
Additional immunosuppression, No. (%)	1 (12.5)	12 (2.5)	13 (2.7)
Number of additional immunosuppressants, median (range)	1 (1–1)	1 (1–3)	1 (1–3)
Anti‐*Pneumocystis* prophylaxis, No. (%)	1 (12.5)	143 (29.9)	144 (29.6)

Abbreviations: CI, confidence interval; IQR, interquartile range; PJP, *Pneumocystis jirovecii* pneumonia; R‐CHOP, rituximab/cyclophosphamide/doxorubicin/vincristine/prednisone; R‐CVP, rituximab/cyclophosphamide/vincristine/prednisone; RICE, rituximab/ifosfamide/ carboplatin/etoposide.

Association of baseline characteristics, laboratory values, chemotherapy, and concurrent immunosuppressive medication use with the occurrence of PJP is presented in Table 3. Stem cell transplant (SCT) occurring any time prior to bendamustine initiation and prior chemotherapy within 1 year of bendamustine initiation were significantly associated with the development of PJP in univariate analyses. Interestingly, concurrent chemotherapy was associated with a decreased risk of being diagnosed with PJP. Steroids and additional, non‐chemotherapy immunosuppressive medications did not have a statistically significant association with the development of PJP infection.

**TABLE 3:** Association of baseline characteristics, laboratory values, chemotherapy, and medication use with occurrence of PJP

Characteristic	Hazard Ratio	95% CI	*p*
Age, years, median (range)	0.98^a^	0.58–1.64	0.93
Male, No. (%)	0.68	0.17–2.74	0.59
Race, No. (%)			
Caucasian	1.02	0.13–132.20	0.99
Other	Reference		
Body Surface Area, m^2^, median (IQR)	1.01^b^	0.79–1.29	0.95
Body Mass Index, kg/m^2^, median (IQR)	0.97	0.86–1.10	0.65
Phase of Diagnosis ‐ Primary, No. (%)	1.30	0.16–168.72	0.85
Charlson Comorbidity Index, median (range)	1.06	0.88–1.28	0.53
COPD, No. (%)	0.68	0.01–5.45	0.78
CKD, No. (%)	0.85	0.11–6.94	0.88
ESLD, No. (%)	33.12	0.25–280.23	0.11
Solid organ transplant, No. (%)	11.74	0.09–94.23	0.22
Stem cell transplant, No. (%)	6.83	1.63–28.69	0.009
Autoimmune Disease, No. (%)	3.05	0.37–24.76	0.30
WBC count, ×10^9^/L, median (IQR)	0.99	0.94–1.04	0.65
Hgb, g/dL, median (IQR)	0.84	0.62–1.13	0.24
ANC, ×10^9^/L, median (IQR)	1.04	0.82–1.31	0.74
ALC, ×10^9^/L, median (IQR)	0.69	0.27–1.76	0.44
Serum Creatinine, mg/dL, median (IQR)	0.91	0.26–3.13	0.88
BUN, mg/dL, median (IQR)	0.96	0.85–1.08	0.51
ALT, units/L, median (IQR)	0.97	0.89–1.06	0.55
AST, units/L, median (IQR)	1.00	0.95–1.04	0.82
ALP, units/L, median (IQR)	1.00^a^	0.90–1.12	0.97
Total Bilirubin, mg/dL, median (IQR)	1.04^b^	0.9601.13	0.34
Direct Bilirubin, mg/dL, median (IQR)	0.99^b^	0.76–1.29	0.95
Albumin, g/dL, median (IQR)	0.98^b^	0.75–1.28	0.86
LDH, units/L, median (IQR)	0.97^c^	0.69–1.38	0.88
Prior chemotherapy within one year, No. (%)	4.55	1.13–18.31	0.033
Cumulative bendamustine dose, mg/m^2^, median (IQR)	0.87^a^	0.71–1.07	0.19
Chemotherapy cycles, median (range)	0.81	0.56–1.17	0.26
Concurrent chemotherapy, No. (%)	0.03	0.01–0.13	<0.001
Steroids received, No. (%)	5.54	0.69–716.01	0.13
Receipt of additional chemotherapy within nine months after bendamustine, No. (%)	1.65	0.41–6.60	0.48
Additional immunosuppression, No. (%)	5.79	0.71–47.09	0.10
Anti‐*Pneumocystis* prophylaxis, No. (%)	0.37	0.05–3.04	0.36

Abbreviations: ALC, absolute lymphocyte count; ALP, alkaline phosphatase; ALT, alanine transaminase; ANC, absolute neutrophil count; AST, aspartate transaminase; BUN, blood urea nitrogen; CI, confidence interval; CKD, chronic kidney disease; COPD, chronic obstructive pulmonary disease; ESLD, end stage liver disease; IQR, interquartile range; LDH, lactate dehydrogenase; PJP, *Pneumocystis jirovecii* pneumonia; WBC, white blood cell.

^a^ Hazard ratios for continuous variable are per 10 unit increase.

^b^ Hazard ratios for continuous variable are per 0.1 unit increase.

^c^ Hazard ratios for continuous variable are per 100 unit increase.

**FIGURE 1:**
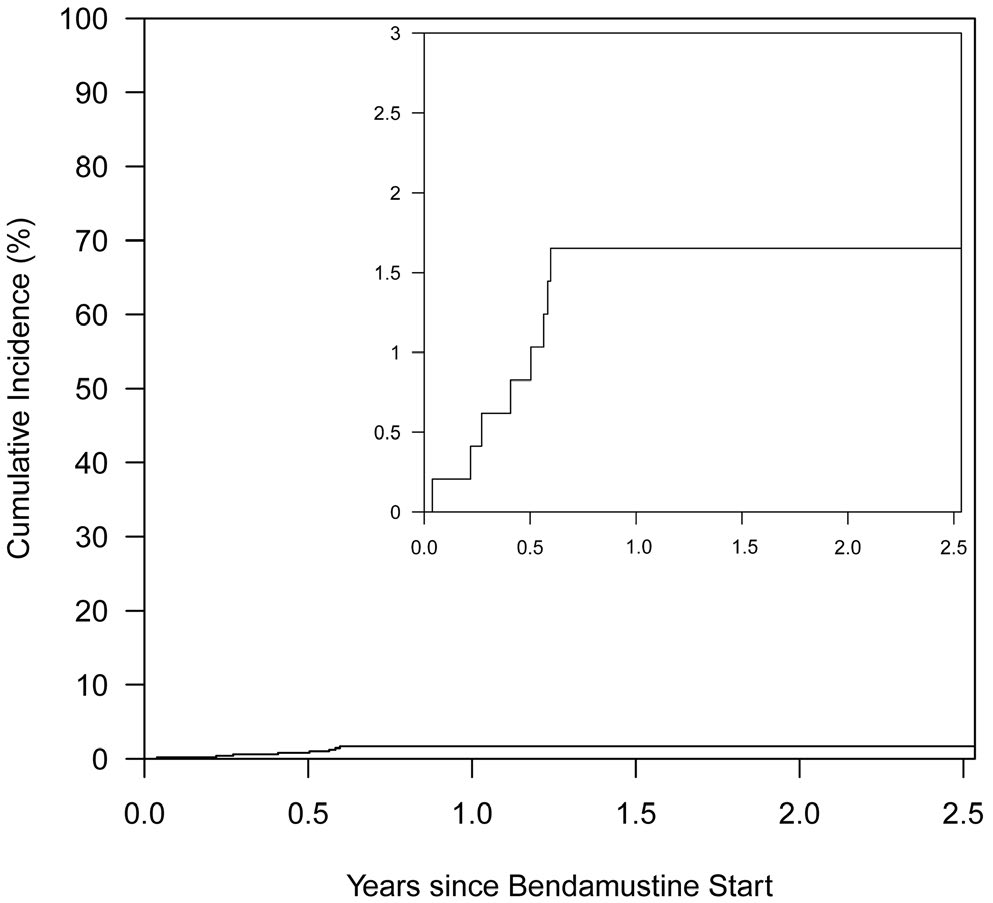
Cumulative incidence of Pneumocystis jirovecii pneumonia from the first administration up to 2.5 years after bendamustine

The cumulative incidence of PJP was 1.7% (95% CI 0.8%–3.3%, at maximum follow‐up of 2.5 years) after the start of bendamustine (n = 8 PJP events overall). Figure 1 depicts the cumulative incidence of PJP from the first administration up to 2.5 years after bendamustine. PJP was diagnosed at a median of 166.5 (range 14–218) days after initiation of bendamustine. Table 4 displays details about the patients diagnosed with PJP. Two patients were admitted to the ICU following PJP diagnosis (patients #4 and #7), and one patient died as a result of PJP (patient #8). Patient #4 was on clinical trial with bendamustine, obinutuzumab, and venetoclax. She presented with severe hyponatremia and bilateral pulmonary infiltrates consistent with a multifocal pneumonia. Over the course of her hospitalization she had witnessed aspiration on tube feeds with worsening hypoxia and tachypnea. She was transferred to the ICU, intubated, and subsequently suffered cardiac arrest with return of spontaneous circulation after 2 min of advanced cardiac life support. Lumber puncture and bronchoscopy demonstrated findings consistent with cytomegalovirus encephalitis and PJP. Patient’s neurological status did not improve, and the patient was compassionately extubated on day eight of her ICU admission. Death was determined to be a result of neurological compromise related to cytomegalovirus infection. Patient #7 was admitted to the ICU for mild PJP and hyperkalemia, which resolved with discontinuation of trimethoprim‐sulfamethoxazole (TMP/SMX) treatment. The patient was switched to atovaquone therapy for PJP and transferred to a medical floor within 48 hours of ICU admission. Patient #8 was diagnosed with PJP and started on intravenous clindamycin and oral primaquine due to a severe sulfa allergy while hospitalized. Due to her malignancy and other comorbidities, she opted to transfer to hospice care without antimicrobial treatment for PJP, and subsequently died within a month of PJP diagnosis.

**TABLE 4:** Characteristics of patients on bendamustine who developed PJP

No.	Age	Sex	Diagnosis	Stage	Other Treatment in Regimen	Line of Therapy	Cumulative Bendamustine Dose (mg/m^2^)	Timing of PJP	Source Tested	Treatment	ICU	Death due to PJP
1	36	F	Hodgkin’s lymphoma	Unstaged	Methylprednisolone	4	760	64 days post‐treatment	BAL	TMP/SMX (IV)	No	No
2	53	F	Angioimmunoblastic T‐cell lymphoma	IV	Methylprednisolone	8	987	59 days post‐treatment	BAL	Atovaquone	No	No
3	60	M	Follicular lymphoma	IV	Rituximab	1	1080	43 days post‐treatment	Sputum	SMX‐TMP (PO)	No	No
4	66	F	Follicular lymphoma	IV	Obinutuzumab Venetoclax	1	1080	78 days post‐treatment	BAL	TMP/SMX (IV)	Yes^a^	No
5	72	M	Multiple myeloma	Unstaged	Dexamethasone	7	100	148 days post‐treatment	BAL	Pentamidine (IV), then TMP/SMX (IV)	No	No
6	72	M	Follicular lymphoma	III	Rituximab	1	540	Cycle 3	BAL	TMP/SMX (PO)	No	No
7	75	M	Peripheral T‐cell lymphoma	IV	None	4	180	Cycle 1	BAL	TMP/SMX (PO), then Atovaquone	Yes^b^	No
8	76	F	Mantle cell lymphoma	IV	Rituximab	1	540	Cycle 3	Sputum	Clindamycin (IV) Primaquine (PO)	No	Yes^c^

Abbreviations: BAL, bronchoalveolar lavage; ICU, intensive care unit; IV, intravenous; PJP, *Pneumocystis jirovecii* pneumonia; PO, oral TMP/SMX, trimethoprim‐sulfamethoxazole.

^a^ Patient presented with severe hyponatremia and bilateral pulmonary infiltrates. Over the course of her hospitalization she had aspiration on tube feeds with worsening hypoxia and tachypnea. She was transferred to the ICU, intubated, and suffered cardiac arrest with return of spontaneous circulation after two minutes of advanced cardiac life support. Lumber puncture and bronchoscopy demonstrated cytomegalovirus encephalitis and PJP. Patient’s status did not improve, and the patient was compassionately extubated on day eight of her ICU admission. Death was determined to be a result of neurological compromise related to cytomegalovirus infection.

^b^ Admission lasting <48 h.

^c^ Patient was started on intravenous clindamycin and oral primaquine due to a severe sulfa allergy while hospitalized. Patient refused to continue antimicrobial treatment for PJP and subsequently died as a result of PJP within a month of PJP diagnosis.

**FIGURE 2:**
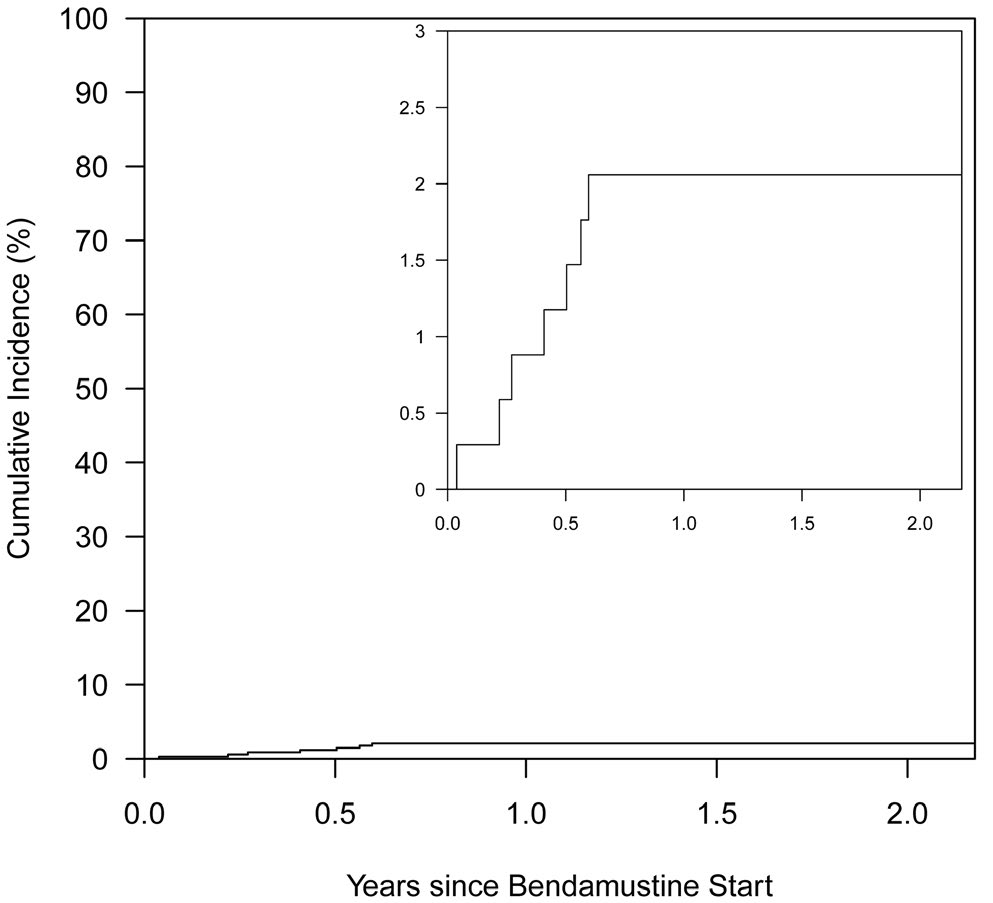
Cumulative incidence of Pneumocystis jirovecii pneumonia in patients who did not receive anti‐Pneumocystis prophylaxis at any time from the first administration up to 2.5 years after bendamustine

Anti‐*Pneumocystis* prophylaxis was prescribed to 144 (29.6%) patients during the treatment and observation period and was at the discretion of the treating physician. The most commonly prescribed agent was TMP/SMX (n = 124, 86.1%), followed by inhaled pentamidine (n = 31, 21.5%). One of the eight patients who developed PJP was prescribed anti‐*Pneumocystis* prophylaxis during bendamustine therapy. This patient (patient #2) developed a mild PJP infection despite receiving inhaled pentamidine as prophylaxis. She was successfully treated with atovaquone without any complications. A month later, the patient underwent allogeneic SCT. Prophylaxis was not significantly associated with a reduction in the development of PJP when compared no prophylaxis (HR 0.37, 95% CI (0.05, 3.04), *p* = 0.36). The Aalen–Johansen method was also used to estimate the cumulative incidence of PJP in the subset of patients who did not receive any anti‐*Pneumocystis* prophylaxis during the study period (Figure 2). In this subset, the cumulative incidence of PJP was 2.1% (95% CI 1.0%–4.3%, at maximum follow‐up of 2.5 years), after the start of bendamustine (n = 7 PJP events overall).

## 4 DISCUSSION

Our study identified a cumulative incidence of PJP diagnosed by PCR of 1.7% in patients on bendamustine therapy. Anti‐*Pneumocystis* prophylaxis was provided at the discretion of the treating physician to 29.6% of patients who received bendamustine‐based regimens during the study period; however, use of prophylaxis was not found to be associated with a decreased incidence of PJP. Prior SCT and prior chemotherapy within 1 year of bendamustine were found to be significantly associated with a diagnosis of PJP. Of the eight patients who developed PJP, two were admitted to the ICU and one death was attributed to PJP.

Anti‐*Pneumocystis* prophylaxis has historically been recommended when the incidence of PJP is greater than 3.5%.^7^, ^8^, ^9^ As our observed incidence and 95% confidence interval bands fell below this threshold, adverse events associated with prophylaxis would likely outweigh the benefit in most patients. We anticipated prophylaxis may be warranted in patients receiving bendamustine based on the known prolonged effect of bendamustine on CD4+ counts; however, this known physiological effect did not result in high rates of PJP in our cohort of patients.^15^, ^16^, ^18^, ^20^ CD4+ data was not readily available for the included patient cohort, limiting further investigation into the explanation for observed PJP rates.

While information on the incidence of PJP in patients on bendamustine is limited, there are several case reports that describe PJP in a variety of immunocompromised patients.^13^, ^14^, ^15^, ^16^, ^17^, ^18^, ^19^ Additionally, while recent clinical trials involving patients receiving bendamustine have reported infectious episodes, few comment specifically on the occurrence of PJP.^30^, ^31^, ^32^, ^33^, ^34^ The phase II CONTRALTO trial, which included patients relapsed/refractory follicular lymphoma, reported PJP in three (6.1%) patients on bendamustine and rituximab plus venetoclax without any cases of PJP in either the bendamustine and rituximab or the venetoclax and rituximab arms for an overall incidence of 3/151 (1.99%) across the three groups.^30^ In an investigation of bendamustine, lenalidomide, and rituximab therapy in patients with mantle cell lymphoma, anti‐*Pneumocystis* prophylaxis was not initially recommended; however, when a patient developed PJP on trial, TMP/SMX was prescribed to patients following a protocol amendment.^31^ Sustained reduction of CD4+ counts occurred in this trial; however, investigators also noted that the addition of prednisone during the induction may have contributed to the high incidence of opportunistic infections, including PJP.^31^ These findings demonstrate a variable PJP rate when prescribing a combination of antineoplastic or immunosuppressive agents with bendamustine. Concurrent chemotherapy, not including steroids, was not associated with an increased risk of PJP in our study. This is potentially explained by the overwhelming majority of patients receiving rituximab as the concurrent treatment with rituximab‐based combination chemotherapy for B‐cell lymphoma. Individual studies and a systematic review and meta‐analysis using various databases have demonstrated a low overall incidence of PJP in the setting of rituximab‐inclusive treatments.^35^, ^36^, ^37^ Due to the retrospective nature of this work, it is impossible to determine if this finding represents selection bias by providers who may have been less inclined to prescribe aggressive, intensive combination chemotherapy to patients with comorbid conditions and a potential higher baseline risk for infectious complications. Univariate analysis incorporating steroids into chemotherapy was impossible as it would include nearly every patient in our cohort and the low overall incidence of PJP in our patient cohort precluded multivariable analysis to assess if there was additional risk conferred by any particular bendamustine‐based combination regimen.

Agents commonly used for prophylaxis have considerable adverse event profiles. One meta‐analysis evaluated TMP/SMX when used for anti‐*Pneumocystis* prophylaxis and found the rate of discontinuation for adverse events to be 15.2%. The rate of discontinuation for serious adverse events was 3.1%.^7^ Avoidance of unnecessary prophylaxis will decrease rates of adverse events, such as rash, myelosuppression, and anaphylaxis, while also reducing the patient’s pill burden. The recommendation against anti‐*Pneumocystis* prophylaxis in this patient population is further supported by our finding that use of prophylaxis was not associated with a significant reduction in risk of developing PJP based on univariate analysis. We did, however, identify certain baseline characteristics that may predispose patients to an increased risk of PJP.

Patients who have undergone SCT are at high risk for opportunistic infections and literature has demonstrated that this risk is not confined to the immediate post‐transplant period.^38^ A recently published investigation compared the incidence of infections occurring two or more years after SCT to matched cancer patients who did not undergo SCT and patients within the general population. PJP was significantly more common in the SCT group.^39^ Prior SCT was found to be associated with development of PJP in our cohort with three of the eight (37.5%) patients diagnosed with PJP having a history of SCT (autologous in patients #1 and #5, allogeneic in patient #2) occurring between 1 and 3 years prior to the initiation of bendamustine. These three patients had extensive chemotherapy treatment histories, including four to eight lines of chemotherapy prior to the initiation of bendamustine therapy in addition to the SCT; however, the number of preceding lines of chemotherapy was not found to be significantly associated with a diagnosis of PJP in our cohort. It may be reasonable to use anti‐*Pneumocystis* prophylaxis in patients on bendamustine‐based regimens with a history of SCT.

The large SEER‐Medicare cohort reported that PJP risk was increased in those who received bendamustine for third‐line therapy, but not first‐ or second‐line treatment.^23^ The low number of PJP events in our cohort precluded us from determining if there was a statistically significant association of PJP with line of therapy; however, prior chemotherapy within 1 year of bendamustine initiation was found to be associated with development of PJP in our cohort. Of the eight patients who developed PJP, four were treated with chemotherapy within the year prior to initiating bendamustine. For these four patients, regimens immediately prior to bendamustine included bortezomib, doxorubicin, and dexamethasone; sorafenib and everolimus; vorinostat, cyclosporine, and dexamethasone; and gemcitabine, dexamethasone, and cisplatin. Recent chemotherapy is another important consideration where anti‐*Pneumocystis* prophylaxis in patients on bendamustine‐based regimens may be warranted.

This study has several limitations worth mentioning with the first being its single‐center, retrospective, non‐randomized design. We included patients over a substantial timeframe to attempt to overcome this limitation and achieve a representative sample. Second, there is the potential to achieve a positive result with *Pneumocystis* PCR in the setting of colonization. While the PCR assay has been designed to minimize this occurrence, we reviewed patient records for clinical signs and symptoms consistent with a diagnosis of PJP to confirm the accuracy of diagnosis. Third, we were unable to determine the dose and duration of steroids used during the study period as well as the temporal correlation to PJP diagnosis. The dose and duration of concomitant steroids should be considered in patients receiving bendamustine therapy to guide the use of PJP prophylaxis; however, we were unable to determine whether PJP prophylaxis would be warranted outside of the standard recommendations for patients on steroids in patients also receiving bendamustine. Finally, a substantial number of patients within our cohort received anti‐*Pneumocystis* prophylaxis. This decision was made at the discretion of individual providers in the absence of specific recommendations for prophylaxis in this patient population. It was not possible to verify that patients were taking prophylactic medications as prescribed.

The cumulative incidence of PJP for patients on bendamustine therapy was 1.7% (95% CI 0.8%–3.3%) falling below the conventional 3.5% threshold for prophylaxis. The low incidence of PJP in this cohort of patients, including in those patients who did not receive anti‐*Pneumocystis* prophylaxis, suggests that most patients on bendamustine therapy do not require routine prophylaxis. Patients with a history of SCT or who received additional chemotherapy within the year prior to bendamustine initiation may benefit from prophylaxis, but an optimal strategy must be further defined.

## CONFLICT OF INTEREST

The authors declare no competing financial interests.

## AUTHOR CONTRIBUTIONS

Conceptualization and methodology: MLR, JNB, CAT, KCM, PKT, ALP; Data curation, formal analysis, and interpretation: MLR, JNB, CAT, KCM, PKT, ALP; Writing – original draft: MLR, JNB, KCM; Writing – review and editing: MLR, JNB, CAT, KCM, PKT, ALP.

## ETHICAL APPROVAL STATEMENT

The study was reviewed by the local Institutional Review Board (IRB) and determined to be exempt from the requirement for IRB approval (IRB #19‐007217, Approval Date: September 20, 2019). After inclusion, an electronic health record (EHR) review was performed in accordance with the ethical standards of the 1964 Declaration of Helsinki with adherence to all relevant regulations of the US Health Insurance Portability and Accountability Act.

## Supporting information

Supplementary Material

## Data Availability

The data that support the findings of this study are available on request from the corresponding author. The data are not publicly available due to privacy or ethical restrictions.

## References

[1] Thomas CF , Limper AH . Pneumocystis pneumonia. N Eng J Med. 2004;350:2487–2498. 10.1056/NEJMra032588 15190141

[2] Carmona EM , Limper AH . Update on the diagnosis and treatment of Pneumocystis pneumonia. Ther Adv Respir Dis. 2011;5(1):41–59. 20736243 10.1177/1753465810380102 PMC6886706

[3] Curtis JR , Yarnold PR , Schwartz DN , Weinstein RA , Bennett CL . Improvements in outcomes of acute respiratory failure for patients with human immunodeficiency virus‐related Pneumocystis carinii pneumonia. Am J Respir Crit Care Med. 2000;162:393–398. 10934059 10.1164/ajrccm.162.2.9909014

[4] Pareja JG , Garland R , Koziel H . Use of adjunctive corticosteroids in severe adult non‐HIV Pneumocystis carinii pneumonia. Chest. 1998;113:1215–1224. 9596297 10.1378/chest.113.5.1215

[5] Sepkowitz KA . Opportunistic infections in patients with and patients without acquired immunodeficiency syndrome. Clin Infect Dis. 2002;34:1098–1107. 11914999 10.1086/339548

[6] Yale SH , Limper AH . Pneumocystis carinii pneumonia in patients without acquired immunodeficiency syndrome: associated illnesses and prior corticosteroid therapy. Mayo Clin Proc. 1996;71(1):5–13. 8538233 10.4065/71.1.5

[7] Green H , Paul M , Vidal L , Leibovici L . Prophylaxis of pneumocystis pneumonia in immunocompromised non‐HIV‐infected patients: systematic review and meta‐analysis of randomized controlled trials. Mayo Clin Proc. 2007;82(9):1052–1059. 17803871 10.4065/82.9.1052

[8] Taplitz RA , Kennedy EB , Flowers CR . Antimicrobial prophylaxis for adult patients with cancer‐related immunosuppression: ASCO and IDSA clinical practice guideline update summary. J Oncol Pract. 2018;36(30):3043–3054. 10.1200/JOP.18.00366 30179525

[9] Fishman JA . Prevention of infection caused by Pneumocystis carinii in transplant recipients. Clin Infect Dis. 2001;33(8):1397–1405. 11565082 10.1086/323129

[10] Knauf WU , Lissichkov T , Aldaoud A , et al. Phase III randomized study of bendamustine compared with chlorambucil in previously untreated patients with chronic lymphocytic leukemia. J Clin Oncol. 2009;27(26):4378–4384. 19652068 10.1200/JCO.2008.20.8389

[11] Bergmann MA , Goebeler ME , Herold M , et al. Efficacy of bendamustine in patients with relapsed or refractory chronic lymphocytic leukemia: results of a phase I/II study of the German CLL Study Group. Haematologica. 2005;90(10):1357–1364. 16219572

[12] Cheson BD , Brugger W , Damaj G , et al. Optimal use of bendamustine in hematologic disorders: Treatment recommendations from an international consensus panel ‐ an update. Leuk Lymphoma. 2016;57(4):766–782. 26592922 10.3109/10428194.2015.1099647 PMC4840280

[13] Klippstein A , Schneider C , Sayer H , Höfflken K . Pneumocystis carinii pneumonia as a complication of bendamustine monotherapy in a patient with advanced progressive breast cancer. J Cancer Res Clin Oncol. 2003;129(5):316–319. 12756557 10.1007/s00432-003-0441-y PMC12162061

[14] Carter SJ , Bernstein SH , Friedberg JW , Barr PM . Pneumocystis jirovecii pneumonia as a complication of bendamustine in a patient receiving bendamustine plus rituximab for marginal zone lymphoma. Leuk Res. 2011;35(11):e223–e224. 21824654 10.1016/j.leukres.2011.07.014

[15] Reinbolt RE , Alam S , Layman R , Shapiro C , Lustberg M . Pneumocystis jiroveci pneumonia in an atypical host. Clin Breast Cancer. 2012;12(2):138–141. 22133356 10.1016/j.clbc.2011.10.003 PMC3498486

[16] Hosoda T , Yokoyama A , Yoneda M , et al. Bendamustine can severely impair T‐cell immunity against cytomegalovirus. Leuk Lymphoma. 2013;54(6):1327–1328. 23072371 10.3109/10428194.2012.739285

[17] Abkur TM , Saeed M , Ahmed SZ , et al. Pneumocystis jiroveci prophylaxis in patients undergoing Bendamustine treatment: the need for a standardized protocol. Clin Case Reports. 2015;3(4):255–259. 10.1002/ccr3.195 PMC4405313 25914820

[18] Ha J , Jung Y , Jung Y , et al. A case of Pneumocystis jiroveci pneumonia after bendamustinebased chemotherapy for refractory diffuse large B‐cell lymphoma. Blood Res. 2016;51(1):61–63. 27104194 10.5045/br.2016.51.1.61 PMC4828532

[19] Henderson J , Morche M , Austein T . Rare side effect of immunochemotherapy with rituximab/bendamustine/prednisolone in a patient with CLL and autoimmune haemolytic anaemia Henderson. Oncol Res Treat Conf. 2017;(September):Band 40, Supplement 3, 154‐155.

[20] Saito H , Maruyama D , Maeshima AM , et al. Prolonged lymphocytopenia after bendamustine therapy in patients with relapsed or refractory indolent B‐cell and mantle cell lymphoma. Blood Cancer J. 2015;5(10):e362. 26495859 10.1038/bcj.2015.86 PMC4635195

[21] NCCN . Prevention and treatment of cancer‐related infections. NCCN Clin Pract Guidel Oncol. 2020;2020(1):1–159.

[22] Cooley L , Dendle C , Wolf J , et al. Consensus guidelines for diagnosis, prophylaxis and management of Pneumocystis jirovecii pneumonia in patients with haematological and solid malignancies. Intern Med J. 2014;44(12):1350–1363. 25482745 10.1111/imj.12599

[23] Fung M , Jacobsen E , Freedman A , et al. Increased risk of infectious complications in older patients with indolent non‐hodgkin lymphoma exposed to bendamustine. Clin Infect Dis. 2019;68(2):247–255. 29800121 10.1093/cid/ciy458 PMC6321852

[24] Arcenas RC , Uhl JR , Buckwalter SP , et al. A real‐time polymerase chain reaction assay for detection of Pneumocystis from bronchoalveolar lavage fluid. Diagn Microbiol Infect Dis. 2006;54:169–175. 16423488 10.1016/j.diagmicrobio.2005.08.006

[25] Caliendo AM , Hewitt PL , Allega JM , et al. Performance of a PCR assay for detection of Pneumocystis carinii from respiratory specimens. J Clin Microbiol. 1998;36(4):979–982. 9542920 10.1128/jcm.36.4.979-982.1998 PMC104672

[26] Wilson JW , Limper AH , Grys TE , et al. Pneumocystis jirovecii testing by real‐time polymerase chain reaction and direct examination among immunocompetent and immunosuppressed patient groups and correlation to disease specificity. Diagn Microbiol Infect Dis. 2011;69:145–152. 21251557 10.1016/j.diagmicrobio.2010.10.021 PMC6855182

[27] Robert‐Gangneux F , Belaz S , Revest M , et al. Diagnosis of Pneumocystis jirovecii pneumonia in immunocompromised patients by real‐time PCR: a 4‐year prospective study. J Clin Microbiol. 2014;52(9):3370–3376. 25009050 10.1128/JCM.01480-14 PMC4313164

[28] Harris PA , Taylor R , Thielke R , et al. Research electronic data capture ( REDCap )— a metadata‐driven methodology and workflow process for providing translational research informatics support. J Biomed Inform. 2009;42(2):377–381. 18929686 10.1016/j.jbi.2008.08.010 PMC2700030

[29] Swerdlow SH , Campo E , Pileri SA , et al. The 2016 revision of the World Health Organization classification of lymphoid neoplasms. Blood. 2016;127(20):2375–2390. 26980727 10.1182/blood-2016-01-643569 PMC4874220

[30] Zinzani P , Flinn I , Yuen S , et al. Venetoclax–rituximab ± bendamustine vs bendamustine–rituximab in relapsed/refractory follicular lymphoma: CONTRALTO [published online ahead of print 12 Aug 2020]. Blood. 10.1182/blood.2020005588. PMC7735159 32785666

[31] Albertsson‐Lindblad A , Kolstad A , Laurell A , et al. Lenalidomide‐bendamustine‐rituximab in patients older than 65 years with untreated mantle cell lymphoma. Blood. 2016;128(14):1814–1820. 27354719 10.1182/blood-2016-03-704023

[32] Cramer P , von Tresckow J , Bahlo J , et al. Bendamustine followed by obinutuzumab and venetoclax in chronic lymphocytic leukaemia (CLL2‐BAG): primary endpoint analysis of a multicentre, open‐label, phase 2 trial. Lancet Oncol. 2018;19(9):1215–1228. 30115596 10.1016/S1470-2045(18)30414-5

[33] Rummel M , Kaiser U , Balser C , et al. Bendamustine plus rituximab versus fludarabine plus rituximab for patients with relapsed indolent and mantle‐cell lymphomas: a multicentre, randomised, open‐label, non‐inferiority phase 3 trial. Lancet Oncol. 2016;17(1):57–66. 26655425 10.1016/S1470-2045(15)00447-7

[34] Eichhorst B , Fink A‐M , Bahlo J , et al. First‐line chemoimmunotherapy with bendamustine and rituximab versus fludarabine, cyclophosphamide, and rituximab in patients with advanced chronic lymphocytic leukaemia (CLL10): an international, open‐label, randomised, phase 3, non‐inferiority trial. Lancet Oncol. 2016;17(7):928–942. 27216274 10.1016/S1470-2045(16)30051-1

[35] Barreto JN , Ice LL , Thompson CA , et al. Low incidence of pneumocystis pneumonia utilizing PCR‐based diagnosis in patients with B‐cell lymphoma receiving rituximab‐containing combination chemotherapy. Am J Hematol. 2016;91(11):1113–1117. 27472910 10.1002/ajh.24499

[36] Jiang X , Mei X , Feng D , Wang X . Prophylaxis and treatment of Pneumocystis jiroveci pneumonia in lymphoma patients subjected to rituximab‐contained therapy: a systemic review and meta‐analysis. PLoS One. 2015;10(4):1–15. 10.1371/journal.pone.0122171 PMC4409297 25909634

[37] Park S , Kang C‐I , Chung DR , et al. Clinical significance of non‐neutropenic fever in the management of diffuse large B‐cell lymphoma patients treated with rituximab‐CHOP: comparison with febrile neutropenia and risk factor analysis. Cancer Res Treat. 2015;47(3):448–457. 25648098 10.4143/crt.2014.034 PMC4506109

[38] Williams KM , Ahn KW , Chen M , et al. The incidence, mortality and timing of Pneumocystis jiroveci pneumonia after hematopoietic cell transplantation: a CIBMTR analysis. Bone Marrow Transplant. 2016;51(4):573–580. 26726945 10.1038/bmt.2015.316 PMC4823157

[39] Foord AM , Cushing‐Haugen KL , Boeckh MJ , et al. Late infectious complications in hematopoietic cell transplantation survivors: a population‐based study. Blood Adv. 2020;4(7):1232–1241. 32227211 10.1182/bloodadvances.2020001470 PMC7160274

