# Supplementary material for: Incidence of Pneumocystis jirovecii pneumonia utilizing a polymerase chain reaction‐based diagnosis in patients receiving bendamustine
Source: Cancer Med. 2021 Jun 22;10(15):5120–30. doi: 10.1002/cam4.4067 (PMC8335812; doi:10.1002/cam4.4067)
Supplement: Supplementary file 1 — Supplementary Material [file CAM4-10-5120-s001.docx]

**Supplemental Table 1. Bendamustine-inclusive combination chemotherapy regimens used as treatment for hematologic malignancy.**

| **Drug** | **Dose** | **Administration details** | **Day(s) of Cycle** |
| --- | --- | --- | --- |
| **Bendamustine. Every 21 to 28 days^#^** | | | |
| Bendamustine | 70 - 120 mg/m^2^ | Intravenous infusion in 0.9% Normal Saline | 1, 2 |
| **Bendamustine + Rituximab. Every 28 days^#^** | | | |
| Rituximab | 375 - 500 mg/m^2^ | Diluted to a concentration of 1 mg/mL and administered as an intravenous infusion at a titratable rate as tolerated | 1 |
| Bendamustine | 70 - 120 mg/m^2^ | Intravenous infusion in 0.9% Normal Saline | 1, 2 |
| **Bendamustine + Brentuximab. Every 21 days** | | | |
| Brentuximab vedotin | 1.8 mg/kg | Intravenous infusion in 0.9% Normal Saline over 30 minutes. For doses of 100 mg or more, dilute in 250 ml. Maximum dose of 180mg. | 1 |
| Bendamustine | 90 mg/m^2^ | Intravenous infusion in 50 ml of 0.9% Normal Saline over 10 minutes for cycles 1 through 6 only | 1, 2 |
| **Bendamustine + Lenalidomide + Dexamethasone. Every 28 days** | | | |
| Dexamethasone | 40 mg | Orally once weekly | 1, 8, 15, 22 |
| Bendamustine | 50 mg/m^2^ | Intravenous infusion in 50 ml of 0.9% Normal Saline over 10 minutes for cycles 1 through 6 only | 1, 2 |
| Lenalidomide | 15 mg | Orally once daily | 1 through 28 |
| **Bendamustine + Prednisone + Bortezomib (BPV). Every 21 days** | | | |
| Bendamustine | 60 mg/m^2^ | Intravenous infusion in 0.9% Normal Saline | 1, 2 |
| Prednisone | 100 mg | Orally once daily | 1, 2, 4, 8, 11 |
| Bortezomib | 1.3 mg/m^2^ | Subcutaneously or intravenous push | 1, 4, 8, 11 |
| **Bendamustine + Prednisone + Thalidomide (BPT). Every 28 days** | | | |
| Bendamustine | 60 mg/m^2^ | Intravenous infusion in 0.9% Normal Saline | 1, 8, 15 |
| Prednisone | 100 mg | Orally once weekly | 1, 8, 15, 22 |
| Thalidomide | 100 mg | Orally once daily | 1 through 28 |
| **Bendamustine + Everolimus. Every 21 days** | | | |
| Bendamustine | 120 mg/m^2^ | Intravenous infusion in 0.9% Normal Saline | 1, 2 |
| Everolimus | 10 mg | Orally once daily | 1 through 21 |
| **Bendamustine + Polalidomide + Dexamethasone + Elotuzumab. Every 28 days** | | | |
| Bendamustine | 120 mg/m^2^ | Intravenous infusion in 0.9% Normal Saline | 1, 2 |
| Pomalidomide | 3 mg | Orally once daily | 1 through 21 |
| Dexamethasone | 40 mg | Orally once weekly | 1, 8, 15, 22 |
| Elotuzumab | 10 mg/kg | Intravenous infusion in 0.9% Normal Saline at a titratable rate as tolerated | Cycle 1 and 2: 1, 8, 15, 22  After cycle 2: 1, 15 |
| **Bendamustine + Obinutuzumab.^#^ Cycles 1 through 6 = Every 28 days; Cycles 7 through 18 = Every 2 months. Cycle 7 starts 8 weeks after Cycle 6.** | | | |
| Obinutuzumab | 1000 mg | Intravenous infusion of 0.9% Normal Saline and administered at a titratable rate as tolerated | Cycle 1: 1, 8, 15  After cycle 1: 1 |
| Bendamustine | 90 mg/m^2^ | Intravenous infusion in 50 ml of 0.9% Normal Saline over 10 minutes for cycles 1 through 6 only | 1, 2 |
| **Bendamustine + Venetoclax. Every 28 days** | | | |
| Bendamustine | 70 - 100 mg/m^2^ | Intravenous infusion in 50 ml of 0.9% Normal Saline over 10 minutes for cycles 1 through 6 only | 1, 2 |
| Venetoclax | 400 mg | Orally with food and water at approximately the same time each day. Initiate therapy with 20 mg  on Day 1, followed by a rapid dose escalation schedule to the recommended daily dose of 400 mg once daily | 1 through 28 |
| **Bendamustine + Rituximab + Polatuzumab. Every 21 days** | | | |
| Rituximab | 375 - 500 mg/m^2^ | Diluted to a concentration of 1 mg/mL and administered as an intravenous infusion at a titratable rate as tolerated | 1 |
| Polatuzumab vedotin | 1.8 mg/kg | Intravenous infusion in 0.9% Normal Saline | 1 |
| Bendamustine | 70 - 90 mg/m^2^ | Intravenous infusion in 0.9% Normal Saline | 1, 2 |
| **Bendamustine + Dexamethasone. Every 28 days** | | | |
| Bendamustine | 120 mg/m^2^ | Intravenous infusion in 0.9% Normal Saline | 1 |
| Dexamethasone | 28 mg | Orally once weekly | 1, 8, 15, 22 |

* Bendamustine-based regimen was used as the foundation along with additional investigational agents or additional chemotherapy medications as part of an IRB-approved clinical trial.
